# Effect of visual marbling levels in pork loins on meat quality and Thai consumer acceptance and purchase intent

**DOI:** 10.5713/ajas.19.0084

**Published:** 2019-05-27

**Authors:** Sawankamol Noidad, Rutcharin Limsupavanich, Suntaree Suwonsichon, Chanporn Chaosap

**Affiliations:** 1 Department of Animal Production Technology and Fisheries, Faculty of Agricultural Technology, King Mongkut’s Institute of Technology Ladkrabang (KMITL), Bangkok, 10520, Thailand; 2 Kasetsart University Sensory and Consumer Research Center, Department of Product Development, Faculty of Agro-Industry, Kasetsart University, Bangkok, 10900, Thailand; 3 Department of Agricultural Education, Faculty of Industrial Education and Technology, KMITL, Bangkok, 10520, Thailand

**Keywords:** Intramuscular Fat, Visual Marbling Level, Pork Loin Quality, Meat Physicochemical Traits, Consumer Palatability Responses, Purchase Intent

## Abstract

**Objective:**

We investigated visual marbling level (VML) influence on pork loin physicochemical traits, consumer palatability responses, VML liking, purchase intent, and their relationships.

**Methods:**

For each of five slaughtering dates, at 24-h postmortem, nine paired Duroc castrated male boneless *Longissimus dorsi* (LD) muscles were categorized into low (LM, score 1 to 2, n = 3), medium (MM, score 3 to 4, n = 3), and high (HM, score 5 to 6, n = 3) VML. Meat physicochemical quality traits and consumer responses (n = 389) on palatability and VML liking, and purchase intent were evaluated. The experiment was in randomized complete block design. Analysis of variance, Duncan’s multiple mean comparisons, and correlation coefficients were determined.

**Results:**

VML correspond to crude fat (r = 0.91, p<0.01), but both were reversely related to moisture content (r = −0.75 and −0.91, p<0.01, respectively). As VML increased, ash (p<0.05) and protein (p = 0.072) decreased, pH and b* increased (p<0.05), but drip, cooking (p<0.05) and thawing (p = 0.088) losses decreased. Among treatments, muscle fiber diameter, sarcomere length, total and insoluble collagen contents, L*, and a* did not differ (p>0.05). Compared to the others, HM had lower collagen solubility percentage (p<0.05), but similar (p>0.05) Warner-Bratzler shear force (WBSF). No differences (p>0.05) were found in juiciness, overall flavor, oiliness, and overall acceptability, but HM was more tender (p<0.05) than the others. Based on VML, consumers preferred MM to HM (p<0.05), while LM was similar to MM and HM (p>0.05). Corresponding to VML preference (r = 0.45, p<0.01), consumers (83%) would (p<0.01) definitely and probably buy MM, over LM (74%), and HM (68%), respectively.

**Conclusion:**

Increasing VML in pork LD altered its chemical composition, slightly increased pH, and improved water holding capacity, thereby improving its tenderness acceptability. Marbling might reduce chewing resistance, as lower collagen solubility in HM did not impact tenderness acceptability and WBSF. While HM was rated as most tender, consumers visually preferred and would purchase MM.

## INTRODUCTION

Pork is the most important red meat consumed in Thailand with an increased consumption of 8.03% from 2013 to 2017 [1]. About 97% of total production was for local consumption, which was approximately 1.4 million tons [1]. Despite the increase in consumption, pork has been described as less healthy and fattier than chicken and beef in consumer’s views [2,3]. While trying to improve carcass lean yield and health image, the pork industry in Thailand is facing the same challenge as the rest of the world, where consumers are demanding a better quality meat. Long-term selection to increase leanness in pork carcasses resulting in lower intramuscular fat (IMF) is at the expense of meat quality and palatability traits [4,5]. Increasing marbling level is, therefore, one strategy for palatability improvement [6]. The effects of marbling and crude fat on meat palatability traits as well as consumer acceptability, however, have long been a debate [7–14]. For example, crude fat was reported to have very low correlation (r = 0.09) to tenderness, but moderately related to juiciness (r = 0.36), flavor (r = 0.22), and overall liking (r = 0.24) [15]. An increase in visual marbling level (VML) of pork loin, on the other hand, was found to correspond to a significant increase in trained sensory tenderness and juiciness, but a non-significant lower slice shear force value [16]. Whereas consumer response to pork loin eating quality was described to be favorable with low Warner-Bratzler shear force (WBSF) and high pH, but for crude fat effect, an improved response was observable only between 1% and 6% levels [12]. Moreover, pork with low to medium VML was more preferred by consumers than that with high VML [9,11]. Consumer hesitation to buy high VML pork was associated with increasing health concerns [2,9,11]. However, in Spain, 55.5% of consumers were identified as “lean loin lovers” and 44.5% as “marble loin lover” [17], indicating market segmentation of VML preference. But at consumption, consumers found that high marbling pork was more palatable [9,17]. In Spain, the IMF threshold level for pork was recommended to be 2.2% to 3.4% [17] but a 1.5% minimum was suggested for Canadian consumers [10]. In addition, consumer preference on pork VML varied among countries due to cultural differences, for example, Japanese, Taiwanese, and Korean consumers were found to prefer more marbled meat [18]. In Thailand, Japanese, Chinese, and Korean cuisines are very popular. The number of Japanese food restaurants, especially, increased continuously in the last decade, with the market share of 20 billion Thai baht (THB) in 2017, increasing approximately 10% from 2016 [19]. Whether this indicated that Thai consumers were familiar with high marbled meat is unsure. At the same time, recommendations for the consumption of animal fat and overall fat seem to be in transition from the last several decades, although suggestions for saturated fat intake are still inconclusive [20]. Recently, in a hierarchical clustering study, among 1,068 raw foods selected from a nutrient database, pork fat was listed as one of the top tenth most nutritious foods for its high contents of B vitamins, minerals, and unsaturated fatty acids [21,22]. All this information may have an impact on Thai consumer attitudes toward fat intake. However, the pork industry still does not know how Thai consumers would respond to pork with different marbling levels both at purchase and at consumption, as well as, their palatability perception as related to meat quality. As consumers are the last step in the production chain, understanding their preferences and purchase intent are necessary [3]. Therefore, we investigated the influences and the relationships of VML (low, medium, and high) of pork *Longissimus dorsi* (LD) muscle on meat physicochemical quality traits, sensory eating quality and VML acceptability, and purchase intent of Thai consumers. The results presented in this manuscript should be of interest not only for Thai swine industry and researchers, but also for international stakeholders who are interested in the pork industry in Thailand.

## MATERIALS AND METHODS

### Animal care and use

Animal care and use committee approval was not obtained for this study, because pork samples were collected after slaughter from a commercial pork packing plant.

### Meat collection and sample preparation

For each of five slaughtering dates, at 24-h postmortem (PM), nine paired boneless pork LD muscles from Duroc castrated male carcasses (110.0±10 kg slaughter weight), produced under the same production system, were selected from the fabrication lines at a commercial pork packing plant (Lopburi, Thailand). Each LD was visually evaluated for surface color and marbling on the 10th and 11th ribs by trained personnel based on the United States (US) National Pork Board [23] standard. Surface color of the LD muscles, evaluated approximately after 10 min of bloom, was between score 3 or 4. VML was categorized into low (LM, score 1 or 2, n = 3), medium (MM, score 3 or 4, n = 3), and high (HM, score 5 or 6, n = 3). After the evaluation, external fat cover on each LD was trimmed off to approximately 2.0-mm-thick. The trimmed LD was individually vacuum-packaged (Ultravac 2100, UltraSource, Kansas City, MO, USA) in a polyvinylidene chloride vacuum bag (B.O.T. Co. Ltd., Bangkok, Thailand) and dipped into hot water (85.0°C±0.5°C) for 2 s. (Ultra shrink 2818, UltraSource, USA). Subsequently, all samples were placed into styrofoam containers with ice (1.4°C±0.8°C, EBI 20, Ebro data logger, Xylem Analytics Inc., Ingolstadt, Germany). All samples were then transported to Meat Technology Research Network Center (MTRNC), Faculty of Agricultural Technology, King Mongkut’s Institute of Technology Ladkrabang (KMITL), Bangkok, Thailand. Upon arrival, vacuum packaged LD muscles were stored in a walk-in chiller (1.6°C±0.3°C). At 48-h PM, ultimate pH was directly measured in triplicate on each LD at the 10th rib, using a pH meter equipped with a spear tip glass electrode (Model SG2-ELK Seven Go, Mettler-Toledo International Inc., Giessen, Germany). The left LD muscles were then assigned for proximate composition and meat quality determinations, while those from the right were used for sensory analysis. LD chops of various thicknesses appropriated for each analysis were then prepared from each muscle. Drip loss and instrumental color measurement were performed on fresh LD slices at 48-h PM. The rest of LD chops were individually vacuum packaged in K-Nylon/Linear Low Density Polyethylene bags (Packmart, Bangkok, Thailand) and kept frozen (−20.0°C±1.0°C) until analysis.

### Measurement of meat physicochemical quality traits

Drip loss was determined from a 2-cm-thick LD chop, which was cut, immediately weighed, and placed on a hook attached inside the plastic container lid. The container was then closed with care to prevent the sample touching any parts of the container and stored at 1.6°C±0.3°C for 48-h before sample removal and reweighed. Drip loss was calculated by measuring weight loss and expressed as a percentage of the initial meat weight [24].

Instrumental color was measured on 2.5-cm-thick LD chops. Each chop for each VML was cut, trimmed off external fat, placed on a styrofoam tray with an absorbent pad, and overwrapped with polyvinyl chloride film (50,000 cm^3^ O_2_/9 μm/m^2^/d/23°C/0% relative humidity). Each tray was displayed in an opened-top refrigerated (3.3°C±1.0°C, EBI 20, Ebro data logger, Xylem Analytics Inc., Germany) display case (Systemform, Bangkok, Thailand). The display case was illuminated with warm white fluorescent lightings with average light intensity of 1,000-lux. At 24-h of bloom, CIE L*, a*, and b* color values [25] were obtained from two different locations on the muscle surface using 2.54-cm-diameter aperture spectrophotometer (MiniScan EZ 45/0 LAV, Illuminant D65, 10° observer, Hunter Associates Laboratory Inc., Reston, VA, USA).

Digital image photography of LD chops representing the three marbling levels were taken using a digital camera (EOS 700D, Canon Inc., Tokyo, Japan), with a setting of ISO-200, F9.0, 1/10 second, macro 0.39 m, and 1.3-ft lens. The camera was held approximately 30 cm away from the unwrapped LD sample presented under a 75-W cool white fluorescent lamp (Philips Electronics Co., Ltd., Samutprakarn, Thailand). These images were later displayed on iPad mini 2 tablets (Apple Inc., Cupertino, CA, USA) for consumer preference evaluation of VML. Using digital images helped reduce problems with color and quality variations due to product handlings or temperature fluctuation during preparation and transportation.

Thawing loss, cooking loss, and WBSF were determined on previously weighed and frozen 5.0-cm thick LD chops. Samples were thawed overnight at 1.6°C±0.3°C, removed from vacuum bags, and reweighed. Thawing loss was calculated by difference and expressed as percentage of initial meat weight before freezing. Each sample was then placed into a 15.2×20.3-cm^2^ high density polyethylene (HDPE) bag (Pantong Thai Pack Co., Ltd, Chonburi, Thailand), heat sealed, and cooked in a water bath set at 80.0°C (NBCT7, Labec, Laboratory Equipment PTY. LTD, Marrickville, Australia). An internal temperature of 71.0°C±0.5°C was monitored with 1.5-mm-diameter flexible fiberglass Type K thermocouple connected with temperature data logger (Testo 176T4, Testo Inc., Lenzkirch, Germany). After cooking, samples were cooled down to 30.0°C±1.0°C in running tap water. Then each sample was removed from the bag and reweighed. Cooking loss was calculated by difference and expressed as a percentage of the initial meat weight before cooking [26]. Each sample was then cut across and along the muscle fibers into eight pieces of 1.0×3.0×1.0-cm^3^ muscle cubes. WBSF was assessed by shearing each muscle cube at a right angle to the fiber direction, using an Instron Universal Testing Machine (Model 2519–104, Instron Corporation, Norwood, MA, USA), equipped with a 50.0-kg load cell and a 200 mm/min cross head speed [27].

Muscle fiber diameter (MFD) was determined from previously frozen 1.5-cm-thick LD chops. Muscle homogenates were prepared following a method described by Tuma et al [28] and observed under the 4x objective using a compound microscope (CX-40, Olympus, Hamamatsu, Japan). Myofibril images were captured with microscope eye-piece camera (Dino-eye AM 7023 B, AnMo electronics corporation, New Taipei City, Taiwan), then measured, and analyzed by the DinoCapture 2.0 software (version 1.5.16C, AnMo electronics corporation, Taiwan). Forty measurements were performed on each of five homogenate droplets per sample. Thus, MFD was determined from an average of 200 measurements per sample. Sarcomere length (SL) data were determined from 1.0×1.0×1.0-cm^3^ muscle cubes, which were treated with series of solutions and calculated from 20 sarcomeres of 20 different muscle fibers according to a formula as previously described [29].

Soluble and insoluble collagen contents were determined with some modifications according to the literature [30]. Briefly, four grams of ground LD was randomly sampled, homogenized with Ringer’s solution at 77°C for 66 min, and centrifuged for 10 min at 2,500 g. The supernatant was hydrolyzed in 12 N HCl and the sediments were hydrolyzed in 6 N HCl for 24 h at 110°C. The analysis was run in duplicate for each LD chop. The amount of hydroxyproline was calculated from standard curve at the absorbance of 550 nm. (Microplate reader; iMark, Bio-Rad Laboratories, Inc., Hercules, CA, USA).

Proximate composition was analyzed according to AOAC International [31] from previously frozen 2.0-cm thick LD chops. Upon analysis, each chop was thawed overnight, cut, and ground for 30 s in a commercial grinder (model RON-FP 315, Ronic, Charollais, France). Moisture content was determined by drying the samples at 105°C in a hot-air oven. Crude protein was determined by the Kjeldahl method, while ether extract was performed by a Soxhlet extraction method. Ash was determined at 550°C overnight in a furnace.

### Consumer sensory evaluation

For affective test, samples were kept frozen and evaluated within 12-d PM. The 3.0-cm thick vacuum packaged LD samples were thawed for 24-h at 1.6°C and cooked at 180°C in electronic broiler ovens (EO-42K, Sharp Corporation, Osaka, Japan). Core temperature of 71.0°C±0.5°C was monitored with 1.5-mm-diameter flexible fiberglass Type K thermocouple connected with temperature data logger (Testo 176T4, Testo Inc., Germany). Each chop was cut into 1.3×1.3×1.3-cm^3^ muscle cubes, placed into double layered HDPE bags insulated in between with paper towels, and kept warm at 54°C in a water bath (NBCT7, Labec, Laboratory Equipment PTY. LTD, Australia) until serving. Consumer tests were performed at various locations in Bangkok and suburb areas. Targeted consumers were recruited conveniently from customers who shopped in the pork sale area of the supermarkets in the shopping malls and from those who worked in the office buildings, university, and research center. Consumers who had no food allergy and liked to eat pork participated. The questions on the ballot and testing procedures were explained. Each consumer was then provided with a toothpick, napkin, and expectorant cup, including unsalted cracker and drinking water for palate cleansing. Two LD muscle cubes from each VML were randomly served in a small white odorless plastic cup labeled with 3-digit random number and covered with an aluminum foil to prevent dehydration. For each testing session, care was taken to ensure that the LD muscle cubes from all the animals in each VML would be served and evaluated. Consumer preference for pork eating qualities, including tenderness, juiciness, oiliness, overall flavor, and overall eating quality, were evaluated using a verbal 9-point hedonic scale, where 9 is extremely favorable, 5 is neither like nor dislike, and 1 is extremely dislike. After eating quality evaluation, consumers visually evaluated their acceptability on the marbling levels of the 3 images of LD chops. Images were coded with different 3-digit random numbers and randomly displayed on the iPad mini 2 tablets (Apple Inc., USA). It was explained to consumers that their judgement should focus only on the marbling levels. VML liking responses were rated using a verbal 9-point hedonic scale. Purchase intent was rated using a verbal 5-point scale, where 5 is definitely buy, 3 is might or might not buy, and 1 is definitely not buy. A reward was provided for each participant.

### Statistical analysis

The experiment was performed in randomized complete block design, where slaughter date was considered as blocking effect, and statistically analyzed using SPSS version 17.0 [32]. Influences of VML (low, medium, and high) on meat physicochemical traits, consumer palatability, VML preference, and purchase intent (% top two box ratings) responses were analyzed using analysis of variance (ANOVA). Means were compared using Duncan’s multiple range test, with a significance at p<0.05. Frequency procedures were used to summarize the demographic and behavior data. From a total of 403 consumers recruited, data from only 389 consumers, who did like to eat pork, were used for statistical analysis. Pearson’s correlation coefficients were analyzed to identify (p<0.05) and to assess the relationships among meat physicochemical quality traits. For the relationships among VML, meat physicochemical traits, consumer sensory eating quality, VML preference, and purchase intent ratings, Spearman’s Rank correlation coefficients were determined.

## RESULTS AND DISCUSSION

### Influences of visual marbling level on physicochemical quality traits of pork loin and their relationships

The influences of VML on physicochemical characteristics of pork LD are present in Table 1. Correlation coefficients among VML and variables for meat physicochemical traits, consumer sensory attributes, and purchase intent of pork LD chops are reported in Table 2. Only the correlation coefficients from the variables, which ANOVA revealed significant influences of VML, are presented. From Table 1, VML affected (p<0.05) moisture, crude fat, and ash contents. The differences (p<0.05) in crude fat percentages among the three VML groups were clearly observed and corresponded to the scores subjectively evaluated (r = 0.91, p<0.01, Table 2). Similar observation (r = 0.71, p<0.01) was previously reported [13]. As expected, when VML increased, moisture content decreased (p<0.05). HM had (p<0.05) lower moisture content than MM and LM, respectively. An increase in marbling level was reported to be involved mainly with increasing in triacylglycerols, the neutral or nonpolar lipids [7,33], resulting in more hydrophobicity. The inverse relationship between fat and moisture contents for meat composition has been well documented [13,14,34–38]. As observed in Table 2, moisture was highly negatively (p<0.01) related to VML (r = −0.75) and crude fat (r = −0.91). Similar relationships between moisture and VML (r = −0.61, p<0.01) and crude fat (r = −0.81, p<0.01) were also reported [13].

From Table 1, the ash content of MM was more than that of HM (p<0.05), whereas LM did not differ (p>0.05) from MM and HM. Previous study [16] reported no statistical difference in ash content (1.15%, 1.20%, and 1.16%, respectively) of uncooked pork loins containing different levels of crude fat (1.96%, 2.50%, and 3.56%, respectively). But crude fat levels in their study were in a lower range compared to our study. Several studies previously reported a decrease in ash content as IMF increased [37,39], but an increase with lean proportion [39]. It is likely that highly marbled pork might contain lower mineral content, depending on the variation in lean, fat, and intramuscular connective tissue (IMCT) proportion and composition. This could be of interest for further investigation, especially if highly marbled pork would be prepared for elderly culinary. Results also showed that the protein content tended (p = 0.072, Table 1) to decrease as VML increased. A significant influence of marbling or IMF in reducing the protein content has been reported [16,38,39].

Ultimate pH was stated to be one of the most prominent factors affecting pork sensory quality [6,13]. In Table 1, pH values of pork LD slightly increased (p<0.05) as VML increased. The pH value of MM was similar (p>0.05) to those of LM and HM. But pH of HM was slightly higher (p<0.05) than that of LM, in agreement with previous study [16]. A tendency toward a slightly higher pH in HM could possibly be due to its association to more oxidative type I or oxidative-glycolytic type IIa muscle fiber. On the other hand, the correlations among pH value and VML and crude fat were low (p>0.05, Table 2), which could be due to the small differences (p<0.05) in pH values among treatments. In contrast, IMF and pH value were reported to be positively correlated (r = 0.32, p<0.05) in Japanese commercial pork. But pork population in their study consisted of IMF ranges from, 0.80% to 7.15% and pH values of 5.71 to 6.29 [14].

The PM shortening of SL or contracted muscle fibers resulted in reduced pork tenderness [40]. From Table 1, however, no influence (p>0.05) of VML on SL and MFD of pork LD was observed. Variation in the amount of collagen in IMCT and its thermal stability contributes to variation in cooked meat tenderness [41]. From Table 1, there were no differences (p>0.05) in total and insoluble collagens among treatments. But there was a tendency (p = 0.061) for MM and HM to have lower amount of soluble collagen compared to LM. The differences (p<0.05) in the amount of soluble collagen content among treatments were clearly observed when they were calculated as percentage of total collagen (Table 1). HM had lower (p<0.05) collagen solubility percentage (CSP) than LM, but both did not differ from MM (p>0.05). From Table 2, CSP had (p<0.05) a low to moderate inverse relationships with crude fat and VML. This indicated that as marbling or crude fat increased, collagen became less soluble or more heat stable. Adipose tissue has been described as a specialized connective tissue, in which the development of both tissues is inseparable [42]. It is likely that IMCT tended to develop and form non-reducible cross-links as marbling accumulated. The CSP also had a low inverse relationship with pH value (p<0.05, Table 2). Thermal stability of IMCT in pork *Semimembranosus* muscle was found to decrease with lower muscle pH [43]. However, for current study, no relationships (r = −0.03 to −0.05, p>0.05, data not shown) among all collagen related parameters and WBSF were found. Thus, the lower collagen solubility in HM did not increase toughness measured by WBSF, as no influence (p>0.05, Table 1) of VML on WBSF was observed. In agreement with Ngapo et al [13] and Cannata et al [16] who reported no influences of marbling levels on instrumental tenderness measurement of pork LD. Numerous contradictory results on the influence of marbling on instrumental tenderness measurement were previously reported [5,10,44].

For water holding capacity (WHC), VML had an influence (p<0.05, Table 1) on drip and cooking losses, and tended to affect thawing loss (p = 0.088). As VML increased, drip and cooking losses decreased (p<0.05). HM had lower (p<0.05) drip loss than MM and LM, but there was no difference (p> 0.05) between MM and LM. Cooking losses of HM and MM were similar (p>0.05), but both had less (p<0.05) cooking loss than LM. Although it could be due to the lower initial moisture content in HM and MM, which resulted in less moisture lost. When calculated based on percentages of moisture content, however, drip losses for HM, MM, and LM were 4.68%, 3.96%, and 2.72%, respectively, whereas cooking losses were 30.71%, 27.90%, and 27.06%, respectively. Thus it indicated that moisture loss tended to decrease as VML increased. Moderate inverse relationships among VML and drip and cooking losses were also observed (p<0.01, Table 2). The influence of VML in reducing drip loss could possibly be explained by the slightly higher (p<0.05) pH value in HM compared to LM. For current study, however, the correlations among pH value and drip and cooking losses were negatively low (p>0.05, Table 2). Similarly, crude fat was moderately inversely correlated to drip and cooking losses (p<0.01, Table 2). Fat might be a barrier for moisture loss during cooking, but this needs further investigation. In contrast, no relationship (p>0.05) between IMF and WHC was reported [14], while pH was more related to drip loss (r = −0.57, p<0.01). Furthermore, drip loss had a low to moderate positive relationship with cooking loss, while both were moderately related to moisture content (p<0.01, Table 2). Previous study [14] also reported a positive relationship between drip and cooking losses (r = 0.34, p<0.01). Therefore, cooking loss tended to be high, when fresh meat had low WHC (more drip loss), but it also depended on the initial moisture content of meat.

For instrumental color measurement at 24-h of bloom before digital image photography, there was no difference (p>0.05, Table 1) in lightness (CIE L*) and redness (CIE a*) values among the treatments. Although VML affected yellowness (CIE b*) value, where HM was slightly more yellow (p<0.05) than MM and LM. The differences were less than 1 unit, which could ensure similarity in bloomed color of the samples used for digital photography. As a result, the CIE b* value was (p<0.05, Table 2) moderately positively correlated with VML and crude fat, but slightly negatively correlated with moisture content. This indicated that when VML increased, the surface 24-h bloom color of pork LD chop tended to be slightly more yellow. Similarly, b* was found to be positively associated to marbling scores (r = 0.42, p<0.001) and crude fat (r = 0.41, p<0.001), but reversely related to moisture content (r = −0.43, p<0.001) [13].

### Demographic profile and pork purchasing and consumption behaviors of consumers

For demographic profile and pork purchasing and consumption behaviors of consumers (n = 389), all respondents were regular pork consumers, consisting of 73.8% females and 26.2% males. Large segment (42.7%) of consumer age was between 36–55 years old, whereas about one-third (31.1%) was between 26–35 years old. These age groups were considered to be working people and expected to be major consumers. There were 14.1% of participants with the age ≤25 years old, who were also major consumers. Some at younger ages in this group might not be pork purchasers themselves, but could have an influence on purchasing decisions of their parents. About 12.1% of participants were >55 years old. Most at this age group tended to be more stable in their careers or already retired. They could have better purchasing ability, but some of them might consume less meat in general. Members of households were primarily 4 to 6 persons (50.4%) and ≤3 persons (44.2%). For educational background, 77.6% of participants had Bachelor’s degree or above. Majority of consumers (78.2%) had household monthly incomes ranged from 7,500 to 85,000 THB, while 13.4% had household incomes of >85,000 THB. Most consumers (79.9%) were regular pork purchasers for their families. Unsurprisingly, most common places where majority of consumers (41.6%) purchased their meat were from wet or traditional market and the stand-alone supermarkets (29.6%). For pork consumption frequency, 96.0% of participants consumed pork at least 2 to 3 times a week. For questions regarding the most important attribute considered when eating pork, which was cooked in Korean or Japanese styles, such as yakiniku or shabu-shabu, 68.2% of consumers considered tenderness, followed by flavor (25.9%), and only 3.5% considered juiciness. Similarly, 74.1%, 17.6%, and 5.9% of consumers considered tenderness, flavor, and juiciness, respectively, as the most important attribute when eating pork cooked as steak.

### Effects and relationships of visual marbling level on consumer responses on pork loin quality and purchase intent

The influences of VML on Thai consumer acceptability on sensory eating quality, the appearance of marbling level (VML liking), and their purchase intents are displayed in Table 3. Purchase intents were reported as top two boxes or percentage of consumers who definitely would buy (score 5) and probably would buy (score 4). VML affected (p<0.05) consumer acceptability on tenderness of pork LD, VML liking, and purchase intent. Consumers gave slightly higher (p<0.05) average tenderness liking score for HM than MM and LM. But MM and LM were similar (p>0.05). The average tenderness liking scores for all treatments were above slightly like to moderately like ratings. Consumers seemed to notice the slight differences in WBSF (0.37 to 0.45 kg), which was not observed statistically (p>0.05, Table 1). Therefore, no relationship (r = 0.16, p>0.05, data not shown) between consumer tenderness acceptability and WBSF was found. It was reported that instrumental tenderness measurements, by WBSF and texture profile analysis, could explain less than 20% of pork loin sensory tenderness variability rated by trained panelists [45]. Affective test, therefore, should be performed in order to investigate consumer perception [45]. In addition, consumer tenderness perception could be confounded with juiciness perception [45]. As observed in this study, tenderness acceptability was (p<0.01, data not shown) highly correlated with juiciness (r = 0.91) and flavor (r = 0.71) acceptability ratings, while moderately related to oiliness (r = 0.63). This indicated that consumers tended to rate the pork sample as more tender, if they found that it was more juicy and flavorful, with acceptable oiliness. In Table 2, tenderness acceptability was slightly correlated (p<0.05) with VML and pH value, indicating that tenderness acceptability tended to increase, when VML and pH value increased, in agreement with Cannata et al [16] and Huff-Lonergan et al [46]. Accordingly, the low to moderate relationships (r = 0.19 to 0.49, p<0.05) between VML and tenderness acceptability were reported [5,16,17,46]. Although, in current study, the relationship between tenderness acceptability and crude fat was positive, it was not significant (p>0.05, Table 2). Marbling seemed to indirectly improved tenderness acceptability through an improved WHC, resulting from a slight increase in pH value. In addition, as fat is soft, it may help reducing chewing resistance, as shown by slightly lower WBSF (p>0.05) in HM.

For other eating sensory attributes, no influence (p >0.05, Table 3) of VML was observed on consumer responses for juiciness, oiliness, overall flavor, and overall acceptability. The difference in IMF of at least 5% was needed for measurable consumer responses on overall acceptability and flavor liking of pork LD to be observed [12]. But this level had no influence on juiciness and tenderness acceptability [12]. Several studies have reported an influence of marbling or IMF on juiciness acceptability by consumers [9,17] or by trained panelists [15,16], or no relationship (p>0.05) [46]. From our observation, however, juiciness seemed to be an attribute which was not easily be discerned by Thai consumers and often be associated with oiliness, as both were found to be moderately related (r = 0.66, p<0.01, data not shown). Interestingly, only small group of consumers (3.5%) considered juiciness as the most important attribute when eating pork slices cooked in Japanese or Korean style foods or cooked as steak in Western style (5.9%). The reason for Thai consumers to put less emphasis on juiciness attribute could be due to cultural differences in food preparation and eating style. Moreover, overall acceptability was highly correlated (p<0.01, data not shown) with tenderness (r = 0.91), juiciness (r = 0.94), overall flavor (r = 0.85), and oiliness (r = 0.73) acceptability responses. While most consumers did not seem to acknowledge juiciness as the most important eating sensory trait, at the point of consumption, juiciness was highly related to overall and tenderness acceptability. While tenderness attribute might be universally well understood by most consumers, juiciness and flavor might be perceived and described differently across the cultures [20]. Moreover, juiciness was moderately associated with thawing loss (r = −0.49, p<0.01, data not shown), whereas no relationships (p>0.05, Table 2) among juiciness and drip and cooking losses were observed. Therefore, careful thawing procedures should be emphasized.

High marbling level observed on pork loin chop surface has been reported to influence consumer acceptability and purchase intent [2,9,11]. By evaluating digital images of pork LD chops, consumer responses showed that VML of MM was much preferred (p<0.05, Table 3) than HM, while LM was rated similarly to MM and HM (p>0.05). By considering the top two box rating percentage, results showed that VML had a significant (p<0.01) influence on consumer purchasing decisions. Percentage of consumers who would definitely and probably buy MM (83.0%) was (p<0.01) more than LM (74.0%), which was also (p<0.01) more than HM (68.4%), respectively. Thus consumer purchasing decisions were somewhat corresponded with their preferences on the appearance of marbling level (p<0.01, Table 2) and were negatively (low to moderate) related to VML and crude fat (p<0.01, Table 2). This meant that high marbling level on pork loin chop surface did cause consumer hesitation to buy. But marbling level did not result in a total rejection on purchasing as they most preferred pork with medium VML. Korean, Taiwanese, and Japanese consumers were reported to prefer meat with moderate amount of VML [18]. Accordingly, pork loins with low [9,11] or medium VML [9] were found to obtain higher (p<0.05) acceptability or purchase intent response than that with high marbling. Health concerns associated with fat content in pork LD was addressed to influence consumer purchasing decision [2,8,9].

## CONCLUSION

Increasing VML in pork LD altered its chemical composition, depending on the variation in proportion and composition of lean, fat, and IMCT. Marbling improved WHC (p<0.05) through the increase in pH value (p<0.05). Despite the improved WHC, consumer juiciness, oiliness, overall flavor, and overall acceptability responses did not differ (p>0.05) among treatments. Juiciness seemed to be an attribute which was not easily be discerned by Thai consumers. Only 3.5% to 5.9% of consumers considered juiciness as the most important attribute when eating pork cooked as shabu-shabu, yakiniku, or steak, while the majority (68.2% to 74.1%) considered tenderness. But overall eating acceptability was strongly associated (p<0.01) with tenderness, juiciness, overall flavor, and oiliness acceptability. Whereas juiciness, overall flavor, and oiliness acceptability responses were highly related (p<0.01) to tenderness acceptability, which was also slightly associated (p< 0.05) with VML and pH value. Marbling seemed to improve tenderness acceptability by improving WHC and possibly reducing chewing resistance. Although CSP decreased (p< 0.05) as marbling increased, it did not impact tenderness acceptability and WBSF. Consumers noticed the slight WBSF differences (0.37 to 0.45 kg), which were not explained statistically (p>0.05). Hence consumer sensory perception did not seem to relate to objective measurement and needs to be investigated specifically. While tenderness of HM was most preferred, consumers visually preferred MM and would definitely and probably buy MM over (p<0.05) LM, and HM, respectively. The impact of consumers’ health food preference on palatability acceptability and purchase intent of pork loin with different VML will be further investigated.

## Figures and Tables

**Table 1:** Effects of visual marbling level (VML), categorized as low (LM, score 1–2), moderate (MM, score 3–4), and high (HM, score 5–6) according to the US National Pork Board [23] standard, on physicochemical quality traits of pork *Longissimus dorsi* muscles

Attributes	VML			SEM	p-value

	LM (n = 15)	MM (n = 15)	HM (n = 15)		
Moisture (%)	73.66^a^	72.79^b^	71.02^c^	0.17	<0.001
Crude fat (%)	2.55^c^	4.03^b^	6.11^a^	0.14	<0.001
Ashes (%)	1.19^ab^	1.25^a^	1.17^b^	0.01	0.043
Crude protein (%)	22.65	22.59	21.98	0.13	0.072
pH value	5.60^b^	5.65^ab^	5.69^a^	0.01	0.021
Muscle fiber diameter (μm)	70.97	64.66	67.10	1.16	0.095
Sarcomere length (μm)	1.79	1.80	1.73	0.02	0.246
Total collagen (mg/g)	3.19	3.15	3.31	0.05	0.405
Insoluble collagen (mg/g)	2.81	2.80	2.96	0.05	0.290
Soluble collagen (mg/g)	0.38	0.35	0.35	0.01	0.061
Collagen solubility percentage	12.01^a^	11.33^ab^	10.64^b^	0.14	0.001
Drip loss (%)	3.45^a^	2.88^a^	1.93^b^	0.18	0.005
Thawing loss (%)	3.67	2.89	2.83	0.17	0.088
Cooking loss (%	22.62^a^	20.31^b^	19.22^b^	0.39	0.004
WBSF (kg/cm^2^)	4.44	4.52	4.07	0.11	0.235
CIE L* or lightness1)	55.50	56.07	57.17	0.32	0.106
CIE a* or redness1)	8.41	8.28	8.45	0.16	0.907
CIE b* or yellowness1)	15.74^b^	15.67^b^	16.32^a^	0.09	0.011

SEM, standard error of mean; WBSF, Warner-Bratzler shear force.

1) Color measurement was performed at 24 h-bloom time in an opened-top display case at 3.3°C±0.6°C.

a–c Means in a row with different letters are significantly different (p<0.05).

**Table 2:** Correlation coefficients among visual marbling levels, meat physicochemical quality traits, consumer responses (n = 389) on tenderness acceptability, and VML liking, and purchase intent of pork *Longissimus dorsi* chops

Items	VML	Moisture	CF	Ashes	pH	CSP	DL	CL	b*	TA	VMLL	PI
VML	1	-	-	-	-	-	-	-	-	-	-	-
Moisture	−0.75**	1	-	-	-	-	-	-	-	-	-	-
CF	0.91**	−0.91**	1	-	-	-	-	-	-	-	-	-
Ashes	−0.22	0.23	−0.19	1	-	-	-	-	-	-	-	-
pH	0.28	0.00	0.11	0.12	1	-	-	-	-	-	-	-
CSP	−0.50**	0.27	−0.33*	0.02	−0.33*	1	-	-	-	-	-	-
DL	−0.46**	0.44**	−0.45**	0.14	−0.00	−0.07	1	-	-	-	-	-
CL	−0.47**	0.49**	−0.44**	−0.04	−0.16	0.18	0.39**	1	-	-	-	-
b*	0.36**	−0.32*	0.40**	−0.15	0.13	−0.10	−0.01	−0.28	1	-	-	-
TA	−0.36*	−0.27	0.28	−0.07	0.37*	−0.29	−0.04	0.01	−0.20	1	-	-
VMLL	−0.09	0.15	−0.14	0.36*	−0.07	−0.05	0.02	0.20	−0.34*	0.16	1	-
PI	−0.38**	0.40**	−0.40**	0.37*	−0.08	0.19	0.23	0.09	−0.37*	−0.35*	0.45**	1

VML, visual marbling levels; CF, crude fat; CSP, collagen solubility percentage; DL, drip loss; CL, cooking loss; TA, tenderness acceptability; VMLL, VML liking; PI, purchase intent.

Pearson’s correlation coefficients were analyzed to assess the relationships among meat physiochemical traits.

Spearman’s rank correlation coefficients were determined to assess the relationships among VML, meat physiochemical traits, and consumer responses.

Levels of significance:

* p<0.05,

** p<0.01.

**Table 3:** Effects of visual marbling level (VML), categorized as low (LM, score 1–2), moderate (MM, score 3–4), and high (HM, score 5–6) according to the US National Pork Board [23] standard, on consumer responses (n = 389) on sensory eating acceptability1), VML liking1), and purchase intent2) of pork *Longissimus dorsi* chops

Items	VML			SEM	p-value

	LM (n = 15)	MM (n = 15)	HM (n = 15)		
Tenderness liking1)	6.58^b^	6.58^b^	6.85^a^	0.05	0.046
Juiciness liking1)	6.33	6.27	6.53	0.05	0.111
Oiliness liking1)	6.13	6.19	6.34	0.06	0.267
Overall flavor liking1)	6.41	6.38	6.46	0.05	0.851
Overall liking1)	6.48	6.49	6.66	0.05	0.290
VML liking1)	6.64^ab^	6.86^a^	6.50^b^	0.05	0.017
Purchase intent2)	74.04^b^	83.03^a^	68.38^c^	0.00	0.000

SEM, standard error of mean.

1) Rated on a 9-point verbal hedonic scale, where 9 = like extremely, 5 = neither like nor dislike, and 1 = dislike extremely.

2) Percentage of consumers who claimed probably and definitely would buy, based on a 5-point verbal scale, where 5 = definitely would buy, 4 = probably would buy, 3 = might or might not buy, 2 = probably would not buy, and 1 = definitely would not buy.

a–c Means in a row with different letters are significantly different (p<0.05).
